# Seroprevalence and associated risk factors for feline panleukopenia virus infection among managed giant pandas in China

**DOI:** 10.1186/s13567-026-01796-w

**Published:** 2026-06-15

**Authors:** Yunli Li, Renyong Jia, Hongwen Zhang, Xin Chen, Jianhong Zeng, Jingchao Lan, Chanjuan Yue, Dongsheng Zhang, Wenjun Huang, Wanjing Yang, Xueyang Fan, Xianzhu Xia, Rong Hou, Na Feng, Songrui Liu

**Affiliations:** 1 Sichuan Key Laboratory of Conservation Biology for Endangered Wildlife, Chengdu Research Base of Giant Panda Breeding, Chengdu, 610081 China; 2 Chinese Academy of Agricultural Sciences, State Key Laboratory of Pathogen and Biosecurity, Key Laboratory of Jilin Province for Zoonosis Prevention and Control, Changchun Veterinary Research Institute, Changchun, 130122 China; 3 College of Veterinary Medicine, Sichuan Agricultural University, Chengdu, 611130 China; 4 College of Life Sciences, Shandong Normal University, Jinan, 272000 China

**Keywords:** Feline panleukopenia virus, giant panda, seroprevalence

## Abstract

Feline panleukopenia virus (FPV) poses a significant threat to carnivores worldwide, with increasing documentation of spillover infections in endangered species, including the giant panda (*Ailuropoda melanoleuca*). Despite the presence of serological evidence indicating historical exposure, contemporary data concerning the prevalence of FPV and the associated risk factors in giant pandas remains limited. This study conducted a comprehensive seroepidemiological survey of 136 giant pandas, representing 262 serum samples, from the Chengdu Research Base of Giant Panda Breeding between 2015 and 2023. The overall seroprevalence of FPV antibodies was 56.87% (149/262, 95% CI 0.51–0.63), with consistently high levels observed across years. Multivariate analysis identified young age and the summer season as protective factors, likely due to maternal-derived antibodies and environmentally mediated reduction in viral persistence, respectively. Although not statistically significant, a transfer history showed a trend toward elevated risk (OR > 1), suggesting that it may still represent a potential risk factor. It is important to note that current vaccination protocols appear to be inadequate in eliciting robust immune responses, thereby underscoring the necessity for the development of tailored vaccines. These findings emphasize the persistent and widespread circulation of FPV in captive giant pandas and advocate for seasonally targeted biosecurity measures, rationalized animal transfer protocols, and enhanced immunization strategies to mitigate infection risks in this vulnerable species.

**Supplementary Information:**

The online version contains supplementary material available at 10.1186/s13567-026-01796-w.

## Introduction

Parvoviruses are a class of non-enveloped, linear, single-stranded DNA viruses that exhibit strong resistance to physical and chemical factors. They maintain infectivity in the environment for extended periods, which facilitates their transmission [1]. The subfamily *Parvovirinae*, which infects vertebrates, includes the pivotal genus *Protoparvovirus*. This genus encompasses several closely related pathogens that pose significant threats to carnivores, such as feline panleukopenia virus (FPV), canine parvovirus type 2 (CPV-2), and mink enteritis virus (MEV), now all included in the unique viral species *Protoparvovirus carnivoran*1 [2]. MEV, CPV-2, and FPV share over 98% genomic similarity, with CPV-2 and MEV considered host-range variants that emerged from an FPV-like ancestor during adaptation to new hosts [3–8].

The highly infectious and lethal nature of FPV is well-documented, and it is rapidly transmissible. Since its discovery, the virus has exhibited a global epidemic trend, ranking among the primary viral pathogens threatening felines. Infection has been observed to induce a range of clinical clinical signs, including vomiting, severe diarrhea, and leukopenia in affected hosts [9–11]. The virus's natural host range has expanded beyond domestic carnivores into wildlife. Spillover infections have been confirmed in multiple endangered wild carnivores, including Siberian Tigers (*Panthera tigris altaica*), cougars (*Puma concolor*), leopards (*Panthera pardus*), and Taiwanese Pangolins (*Manis pentadactyla pentadactyla*) [12–17]. This pattern underscores its significance at the domestic animal–wildlife interface, where pathogen exchange threatens conservation efforts.

The giant panda (*Ailuropoda melanoleuca*) as a species of particular significance in the context of global biodiversity conservation, is also vulnerable to this threat. Despite the growth of captive populations resulting from conservation efforts, increased population density and frequent individual transfers would heighten the risk of viral introduction and spread [18, 19]. Parvoviruses (including canine parvovirus, CPV and FPV) have become significant pathogens, posing a grave threat to the health of giant pandas. These viruses can cause gastrointestinal distress and even result in mortality [18, 20, 21]. Serological surveys conducted since the 1980 s have indicated the presence of CPV/FPV antibodies in a proportion of both captive and wild giant pandas, thereby suggesting a history of widespread past infections [22–24]. Alarmingly, recent studies have reported the isolation of giant panda-derived parvovirus strains (including FPV and CPV-2) carrying mutations in the VP2 protein and evidence of recombination between CPV-2 and FPV, suggesting ongoing viral evolution and potential for sustained transmission within giant panda populations [20, 25–27]. Furthermore, the presence of recombinant viruses between CPV and FPV has been detected [28], suggesting potential for sustained transmission and adaptive evolution within giant panda populations. Of particular concern are two recent fatal cases of young giant pandas exhibiting severe gastrointestinal symptoms due to FPV infection [20]. These cases underscore the virus's grave threat to the population's health.

Presently, systematic surveys on parvovirus (particularly FPV) antibody prevalence within giant panda populations are limited, resulting in a paucity of data on recent infection patterns and risk factors. Therefore, the present study conducted a seroepidemiological investigation based on blood samples continuously collected from the Chengdu Research Base of Giant Panda Breeding (CRBGPB), China between 2015 and 2023. The aim of the present study was to evaluate the prevalence of FPV antibodies and the associated risk factors, thereby providing essential data for the development of scientifically sound vaccination strategies and biosecurity measures.

## Materials and methods

### Sample collection

A total of 262 serum samples were obtained from 136 giant panda individuals (81 females and 55 males) at the Chengdu Research Base of Giant Panda Breeding (CRBGPB) in China between 2015 and 2023. All samples were collected during routine annual health check-ups of the animals, none of the sampled pandas showed clinical signs of FPV at the time of collection. Within the same calendar year, each individual contributed only one sample. Of these, 65 individuals were sampled across two or more different years (range: 2–5 time points; Additional file 1). None of the giant pandas in the study had received vaccination against FPV during this period. Blood samples were obtained via venipuncture. The whole blood was then incubated at 37 °C for one hour and refrigerated overnight at 4 °C before being centrifuged at 3000 rpm for 20 min. The serum was carefully aspirated and inactivated at 56 °C for 30 min, and stored at −20 °C for future use. Background information including the sampling date, the age, sex, and transfer history of animals was recorded. All samples collection were approved by the Institutional Animal Care and Use Committee of the Chengdu Research Base of Giant Panda Breeding protocol (protocol #2019008).

### Serological analysis

Hemagglutination inhibition (HI) assays were performed to detect the serum antibodies against FPV. The serum samples were two-fold serially diluted and mixed 1:1 with PBS containing 8 HA units/25 μL of virus antigen (preserved by the Changchun Veterinary Research Institute, Chinese Academy of Agriculture Sciences). The HI titer was expressed as the highest serum dilution ratio that completely inhibits the viral HA of 1% pig red blood cells by 8 HA units/25 µL after one hour incubation at 37 ℃. An HI titer of 1:20 or more was considered positive [29].

### Statistical analysis

The individuals sampled each year were not fixed, and some individuals were sampled repeatedly across different years (Additional file 1). Consequently, the data analysis strategy was as follows: Firstly, for descriptive statistics of the population and annual data (seropositivity rates), we utilized all available test data (n = 262) to comprehensively present the distribution characteristics of the samples. Secondly, to satisfy the fundamental assumption of data independence in statistical inference, all association analyses (such as using binary logistic regression to assess the influence of factors like gender, age (defined as young: 0–1.5 years, sub adult: 1.5–5 years, adult: 5–20 years, and old: > 20 years), season, and transfer history) were based on an independent dataset. This dataset was constructed by retaining only the most recent test result for individuals with duplicate records, ensuring each unique individual contributed a single data point. The effective sample size for inferential statistics was thus n = 136. Thirdly, to explore the longitudinal dynamics of FPV serostatus in repeatedly sampled individuals, we analyzed a subset of 18 animals that had been sampled across at least four different years. These individuals were stratified by transfer history (Group B with transfer history, n = 10; Group A without transfer history, n = 8). The complete sampling history and serostatus for these 18 individuals are provided in Additional file 2.

The seroprevalence of FPV was calculated as the proportion of seropositive samples relative to the total number tested, with 95% confidence intervals (CI) estimated using the Wilson score method. Associations between seropositivity (a binary outcome variable) and potential risk factors—including sex, age group, season, and transfer history—were first assessed using Pearson’s chi-square tests or Fisher’s exact tests, as appropriate. Variables showing an association with *p* < 0.05 in these univariate analyses were included in the multivariable binary logistic regression model. The final model was evaluated for goodness-of-fit using the Hosmer–Lemeshow test. Collinearity among independent variables was examined via correlation analysis to ensure stability and reliability of the model estimates. All statistical analysis were run in R version 4.5.1.

## Results

### Seroprevalence and temporal trends of FPV in giant pandas

**Figure 1:**
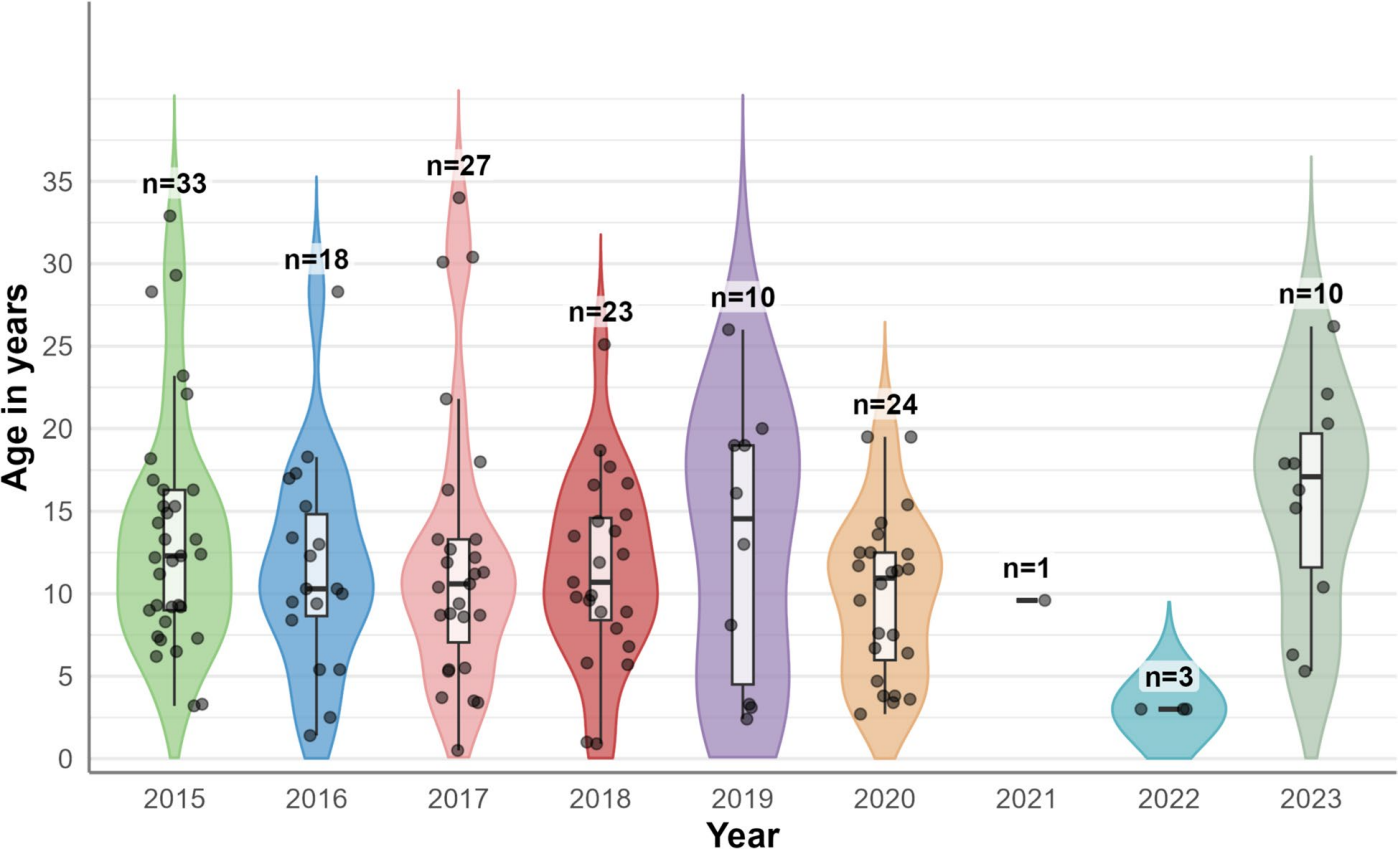
**Temporal distribution of feline panleukopenia virus (FPV) seroprevalence in the managed giant panda population from 2015 to 2023**

Based on the total number of serum samples tested (n = 262, sample-based analysis), The overall seroprevalence of FPV antibodies was 56.87% (149/262, 95% CI 0.51–0.63). The annual seroprevalence exhibited fluctuating trends, with relatively low seroprevalence in 2021 and 2022 at 20.0% (95% CI 0.04–0.62) and 16.7% (95% CI 0.06–0.39) respectively, while other years demonstrated higher antibody positivity rates (above 44.2%), In 2020, the seroprevalence rate even reached 80.0% (24/30, 95% CI 0.63–0.90). Detailed annual testing volumes and positivity rates are presented in Table 1 and Figure 1.

**Table 1:** **Annual seroprevalence of feline panleukopenia virus (FPV) in the managed giant panda population in CRBGPB**

Years	No. of positive	No. of Tests (n = 262)	Seroprevalence% (95% CI*)
2015	33	50	66.0(0.52–0.78)
2016	18	32	56.3(0.39–0.72)
2017	27	45	60.0(0.45–0.73)
2018	23	52	44.2(0.32–0.58)
2019	10	15	66.7(0.42–0.85)
2020	24	30	80.0(0.63–0.90)
2021	1	5	20.0(0.04–0.62)
2022	3	18	16.7(0.06–0.39)
2023	10	15	66.7(0.42–0.85)
total	149	262	56.87(0.51–0.63)

^*^CI: Confidence Interval, calculated using the Wilson score method.

Each individual contributed one sample per year, the annual seroprevalence is sample-based (descriptive).

The total number of tests includes multiple samples from the same individuals across different years.

### Factors associated with FPV seropositivity

The results of the bivariate analysis, based on a dataset of 136 giant pandas' independent serum sample, are presented in Table 2. The model revealed that sex did not have a significant effect on the prevalence of FPV (*P* > 0.05). However, age, season, and transfer history were identified as significant predictors of FPV seropositivity (*P* < 0.05).

**Table 2:** **Bivariate analysis of associations between potential risk factors and FPV seroprevalence in managed giant panda population in CRBGPB**

Variables			No. positives/overall (n = 136)	Seroprevalence		95% CI	Statistic
Sex	Male		26/55	60.50%		0.34–0.60	χ^2^ = 2.315 df = 1 *P* = 0.128
	Female		49/81	47.30%		0.50–0.70	
Age	Young		1/17	5.90%		0.01–0.27	χ^2^ = 32.787 df = 3 *P* < 0.001***
	Sub adult		15/39	38.50%		0.25–0.54	
	Adult		49/68	72.10%		0.60–0.81	
	Old		10/12	83.30%		0.55–0.95	
Season	Spring		18/33	54.50%		0.38–0.70	χ^2^ = 11.661 df = 3 *P* = 0.009**
	Summer		10/32	31.20%		0.18–0.49	
	Autumn		22/36	61.10%		0.45–0.75	
	Winter		25/35	71.40%		0.55–0.84	
Transfer history	Yes		50/78	64.10%		0.53–0.74	χ^2^ = 5.930 df = 1 *P* = 0.015**
	No		25/58	43.10%		0.31–0.56	

^*^P ≤ 0.05: Statistically significant.

**P ≤ 0.01: Highly statistically significant.

***P ≤ 0.001: Extremely statistically significant.

**Figure 2:**
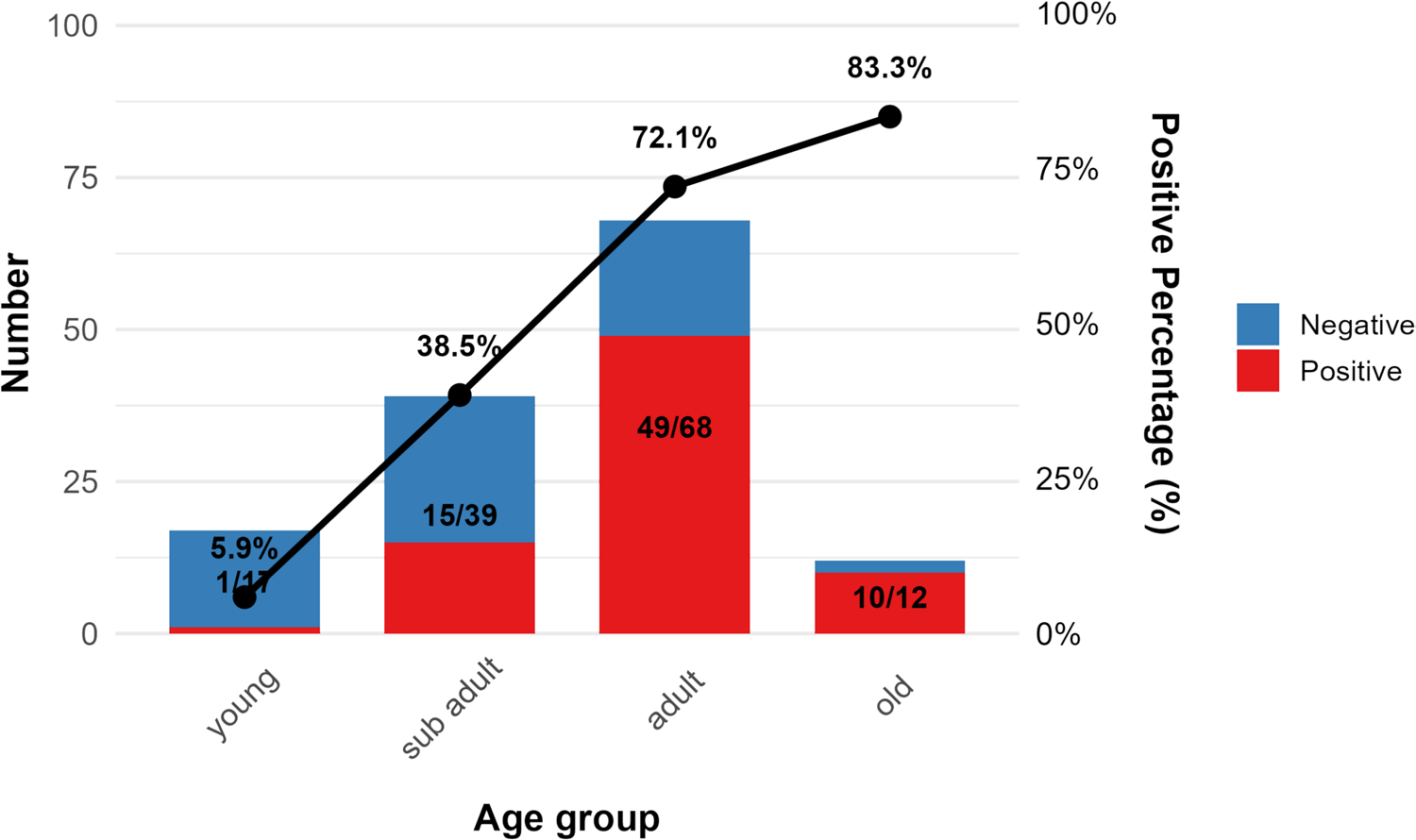
**Age-specific patterns of FPV seroprevalence in giant pandas. **Bars represent the proportion of seropositive (red) and seronegative (blue) individuals within each age group. The line graph depicts the increasing trend in seroprevalence rate across age categories

**Figure 3:**
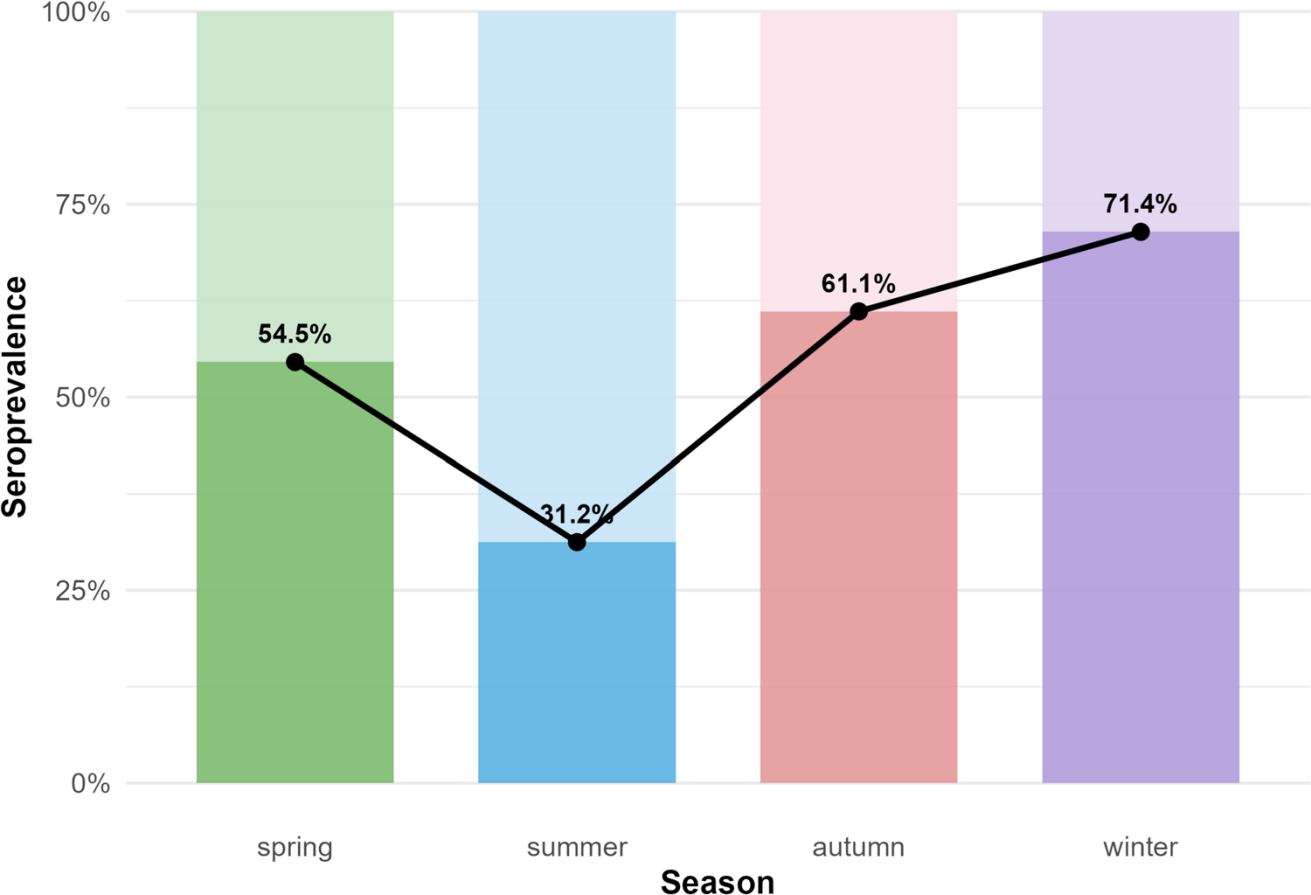
**Seasonal variation in FPV seroprevalence in the giant pandas**. The background color intensity reflects the proportion of seropositive (dark) and seronegative (light) individuals across spring, summer, autumn, and winter

**Figure 4:**
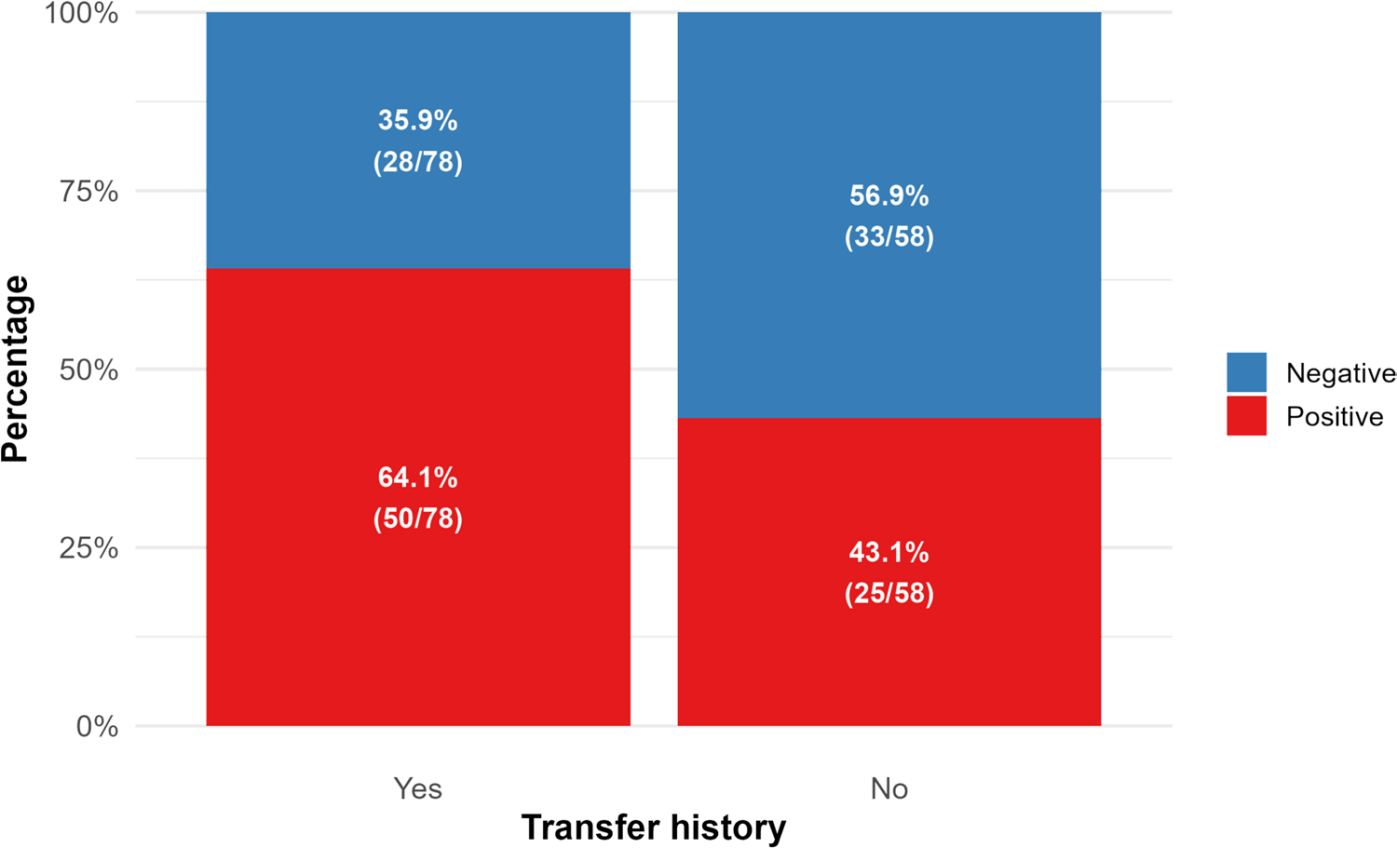
**Association between transfer history and FPV seroprevalence in giant pandas**. Red and blue segments denote seropositive and seronegative individuals, respectively, grouped by whether they had a transfer history between different locations (including facilities and wild origins)

Seroprevalence of FPV antibodies exhibited a significant age-dependent increase, with the highest rate observed in the old (83.3%, 95% CI 0.55–0.95), followed by adults (72.1%, 95% CI 0.60–0.81), sub adults (38.5%, 95% CI 0.25–0.54), and cubs (5.9%, 95% CI 0.01–0.27) (Table 2; Figure 2). A pronounced seasonal variation in seropositivity was also identified. The rate was highest in winter (71.4%, 95% CI 55.1–84.0), followed by autumn and spring, with summer showing the lowest prevalence (31.2%, 95% CI 0.18–0.49) (Table 2; Figure 3). Furthermore, individuals with a transfer history demonstrated a significantly higher seroprevalence (64.1%, 95% CI 0.53–0.74) compared to their non-transferred counterparts (43.1%, 95% CI 0.31–0.56) (Table 2; Figure 4).

Variables with a p-value < 0.1 in this initial screening (namely, age group, season, and transfer history) were included in the multivariable logistic regression model. The results of the final model are presented in Table 3. The model was statistically significant (χ^2^ = 48.498, *p* < 0.001), and the Hosmer–Lemeshow test indicated good model fit (χ^2^ = 4.211, *p* = 0.838). Analysis revealed that young age and the summer season were independent protective factors for positive results (*P* < 0.01). Compared with the elderly group, the risk of a positive result was significantly reduced by 99.2% in the young group (OR < 1, *P* < 0.01). Although a transfer history was positively associated with the risk of a positive result (OR = 1.465), this association was not statistically significant (95% CI 0.604–3.550, *P* = 0.398). Detailed results are presented in Table 3.

**Table 3:** **Risk factors for FPV seropositivity identified by multivariable binary logistic regression analysis**

Factors		B	S.E	OR	95% CI	*P* value
Age	Young	−4.835	1.386	0.008	0.001–0.120	< 0.001
	Sub adult	−1.811	0.890	0.164	0.029–0.937	0.042
	Adult	−0.648	0.879	0.523	0.093–2.927	0.461
	Old	–	–	–	–	–
Season	Spring	−1.688	0.666	0.185	0.050–0.682	0.011
	Summer	−2.013	0.694	0.134	0.034–0.521	0.004
	Autumn	−1.052	0.697	0.349	0.089–1.369	0.131
	Winter	–	–	–	–	–
Transfer history	Yes	0.383	0.452	1.465	0.604–3.550	0.398
	No	–	–	–	–	–

“-” baseline; B: unstandardized regression coefficient; S.E.: standard error of the coefficient; OR: Odds Ratio; CI: Confidence Interval.

### Longitudinal dynamics of FPV serostatus in repeatedly sampled individuals

**Figure 5:**
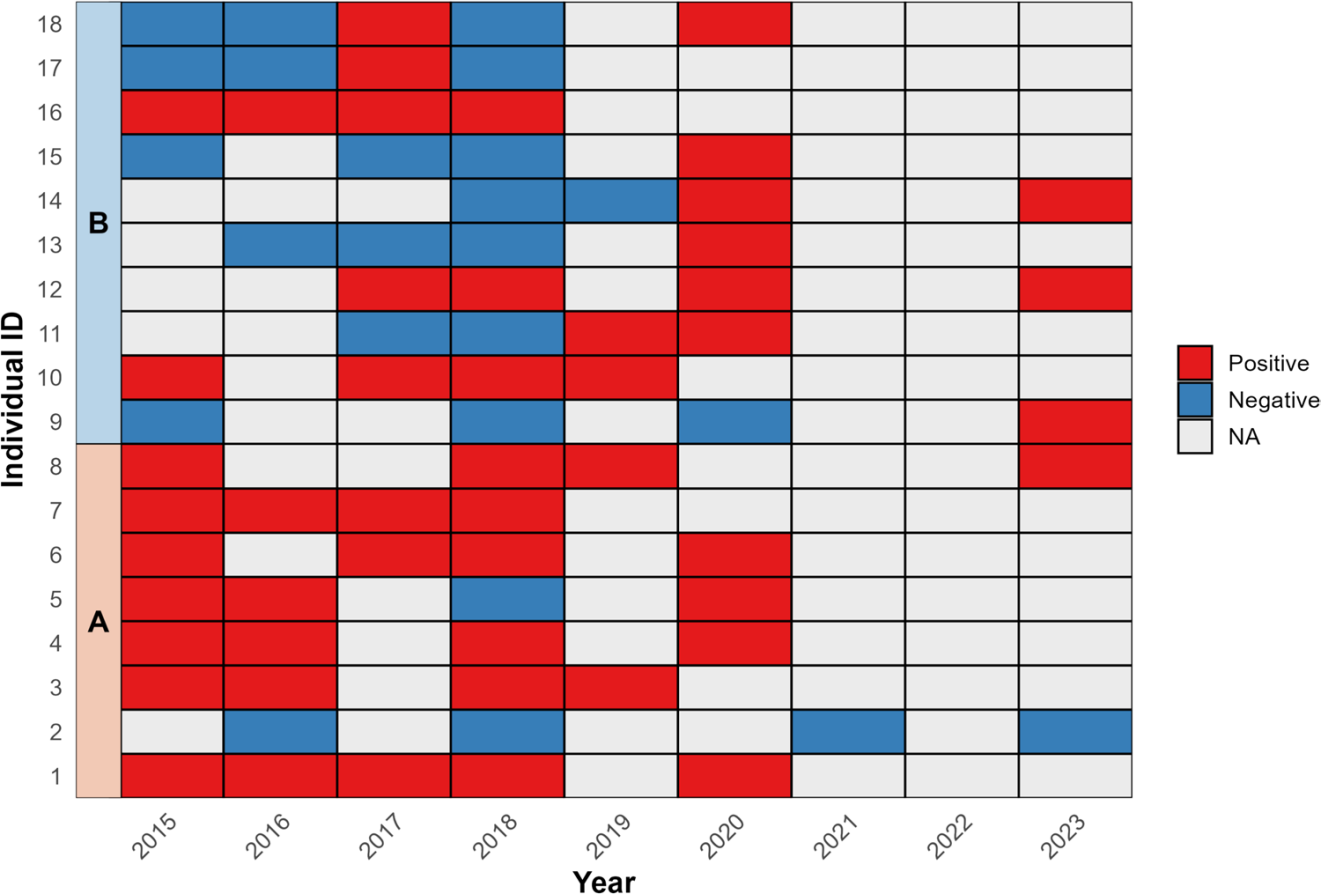
**Longitudinal dynamics of FPV serostatus in 18 giant pandas with repeated sampling (2015–2023)**. Heatmap showing serostatus of individuals with (n = 10, light blue bar on left) and without (n = 8, orange bar on left) transfer history. Red squares indicate seropositive; blue squares indicate seronegative; gray squares indicate no sample available in that year

To further explore the temporal dynamics of FPV infection, we analyzed a subset of 18 individuals that had been sampled repeatedly across different years (Additional file 2). Strikingly, the longitudinal patterns differed between the group A and group B (Figure 5). Among individuals with transfer history (group B), 70% (7/10) exhibited seroconversion from negative to positive after returning to the captive population. These seroconversion events were not temporally aligned with the transfer events themselves; rather, they occurred in subsequent years following reintroduction into the captive population. The remaining 30% (3/10) were persistently seropositive across all time points. Among individuals without transfer history (group A), the majority (75%, 6/8) were persistently seropositive across all sampling time points. Of the remaining two individuals, one was consistently seronegative throughout the study period, and the other was seropositive at all time points except for a single negative result in one intermediate year. No individual in the group A exhibited seroconversion from negative to positive during the study period.

## Discussion

FPV infection primarily causes severe clinical signs such as diarrhea and myocarditis in animals and is relatively common in felines [10]. In recent years, there have been successive reports of giant pandas contracting FPV and exhibiting clinical signs or even dying, posing a serious threat to the health of the giant panda population [19, 20]. In our study, the overall seroprevalence of FPV antibodies was 56.87% (149/262, 95% CI 0.51–0.63), with annual seroprevalence exhibiting remaining high. This warrants particular attention.

As observed, young age served as a protective factor against FPV seropositivity (99.2% risk reduction), an unexpected finding given that young animals are typically considered more vulnerable to parvovirus infection [6]. It is particularly noteworthy that in kittens, the course of the disease is frequently acute, often resulting in high mortality (> 90%) [30]. However, the low seropositivity in giant panda cubs can be explained by two complementary factors: prolonged passive immunity from maternal-derived antibodies (MDA) and reduced exposure risk due to intensive nursery management. In kittens, although maternal antibodies cannot completely prevent infection, they can interfere with active seroconversion, and their effect varies depending on the quantity, quality, and duration of colostrum intake [31]. Proteomic analysis of giant panda milk has revealed that the colostrum-to-mature milk transition lasts approximately 30 days, with persistent IgG levels [32]. Young giant pandas acquire high levels of MDA through maternal milk, which provides passive immune protection, making them less susceptible to infection or clinical symptoms in early life [31–33]. Another potential explanation for the low seropositivity in cubs lies in differences in exposure history. Young giant pandas exhibit more circumscribed activity patterns than adults, presumably due to their confinement to nurseries or rearing rooms. Their environment is characterized by relative uniformity and cleanliness. Consequently, their exposure to viruses is significantly lower than that of adults, which frequently move between enclosures or have been transferred to other facilities. Even after passive immunity wanes, young dogs or cats remain susceptible to parvovirus infection [34, 35]. The sharp increase in seroprevalence observed from the young group to the sub adult, adult, and old groups (from 5.9% to 38.5%, 72.1%, and 83.3%, respectively) suggests a critical window of susceptibility—the period when maternal antibody levels decline to sub-protective levels, before active immunity is acquired through natural exposure or vaccination. This finding provides empirical evidence for the optimization of prophylactic strategies, such as determining the optimal timing for primary vaccination, in this endangered species.

Another protective factor evident in our results is the summer season. The seroprevalence of FPV antibodies was lowest in summer (31.2%, 95% CI 0.18–0.49) and highest in winter (71.4%, 95% CI 0.55–0.84), a pattern consistent with recent epidemiological studies in Egypt [36]. FPV, a non-enveloped virus, exhibits exceptional environmental resilience. However, its survival time is significantly reduced under high temperatures, dry conditions, and ultraviolet radiation exposure [37]. Summer climate conditions are less conducive to the persistence of the virus in outdoor environments compared to other seasons, thereby reducing transmission risks. Winter conditions with lower intensity and shorter duration of sunlight favor prolonged environmental survival of the virus [36]. It is worth noting that the seasonality of FPV varies across countries; for example, it is more common during the summer in Australia and the United States, a difference influenced by variations in the peak of the estrous cycle [36, 38, 39]. The reproductive cycle of the giant panda is characterized by seasonal breeding in the spring and summer months, with parturition occurring in summer [40]. The number of cubs born in the summer with high levels of maternal antibodies may result in a lower overall seroprevalence rate during that season. Additionally, behavioral patterns of captive giant pandas may reinforce the seasonal trend. During hot summer months, giant pandas typically reduce outdoor activity and spend more time indoors, where air conditioning and reduced contact with contaminated soil or fomites may lower their exposure to the virus [41]. These combined ecological, physiological, and behavioral factors explain the observed seasonal variation in FPV seroprevalence. From a conservation management perspective, our findings highlight the winter months as a critical period for enhanced biosecurity measures, including intensified disinfection of enclosures and careful monitoring of susceptible individuals.

Our study also identified the factor of transfer history: although the OR was greater than 1, it was not statistically significant. The wide confidence interval (0.604–3.550) indicates high data uncertainty, neither confirming nor ruling out its potential as a risk factor. Our findings indicate that transfer history is not a direct source of FPV infection, but rather a marker for relocation into a multi-host pathogen interface. FPV is known to circulate endemically in cats and exhibits a remarkable capacity for cross-species transmission to a broad range of wildlife [12–17]. Stray cats sharing the same habitats play a key role in the cross-species transmission of FPV to captive wildlife [12, 42], and furthermore, the FPV strain recently detected from giant pandas was considered to originate from an FPV spillover event involving stray cats in the same habitat [19]. This evidence indicates that the captive giant panda environment shares viral ecology with nearby stray dogs and cats, creating a persistent risk of pathogen spillover at the interface between wildlife and stray animals. Consequently, the overall high seroprevalence (56.87%) observed in managed giant panda population reflects cumulative exposure within such a multi-host setting. Individuals without transfer history are raised within this complex interface from an early age, leading to persistent seropositivity (75% in the longitudinal cohort). In contrast, transferred individuals are reintroduced into a complex environment containing stray dogs and cats, giant panda populations, and red panda populations-an environment where there is a risk of FPV cross-species transmission. This explains our longitudinal observation that 70% of transferred individuals seroconverted after their return, whereas non-transferred residents predominantly remained persistently positive. Therefore, from a conservation management perspective, enhanced biosecurity during animal transfers, including pre- and post-arrival quarantine and serological screening, should be prioritized as a general practice to mitigate pathogen introduction at these critical wildlife-domestic animal interfaces.

It has been demonstrated that FPV exhibits a predilection for replication in rapidly dividing cells. Kittens have been shown to be most susceptible to infection; however, cats of all ages are at risk, especially if they are unvaccinated and live in high-density areas [43]. Research findings on the immunogenicity of standard vaccination protocols in kittens indicate that many cats fail to achieve effective protection through these methods [44]. This phenomenon has also been observed in the context of giant pandas. The antibody titres and distribution patterns in vaccinated giant pandas (using live multivalent vaccines developed for dogs, including CDV, CPV, CAV-1, CCV, canine parainfluenza virus (CPIV), and rabies virus) did not meet the expected efficacy standards for effective vaccines [22, 29]. Pre-breeding booster vaccinations in queens are an effective strategy to maximize the transfer of maternally derived antibodies (MDA), which are critical for protecting kittens from fatal infections during the neonatal period [45]. Consequently, the development of effective vaccines tailored for wildlife is imperative for future conservation efforts.

This study analyzed associations based on existing data. Results may be influenced by the lack of comprehensive, continuous testing of all individuals and the presence of unmeasured confounding factors such as individual stress levels or immune status. Furthermore, seropositivity indicates only exposure history; while the infection was likely natural, whether it was recent or past cannot be determined from seropositivity alone. For young individuals, monitoring maternal antibody decay patterns can determine optimal primary vaccination timing to avoid infection during the “window period” (when MDA fades but vaccine antibodies have not yet developed) [30]. Regarding animal transfer history, although the statistical significance is not high, the trend of OR > 1 warrants vigilance, strict quarantine and pathogen mitigation protocols should be established. Future research should also combine nucleic acid testing with antibody detection, and perform virus isolation and gene sequencing to track circulating strains and viral mutations.

## Conclusions

This study reveals a high and persistent seroprevalence of FPV in the managed giant pandas, underscoring its role as an endemic pathogen with significant conservation implications. Young age and the summer season were identified as independent protective variables, likely mediated by passive immunity and environmentally reduced viral survival, respectively. Although not statistically significant, the suggested increased risk associated with animal transfer history reflects how management activities may influence disease dynamics through stress or introduction of new exposure sources. The inadequate efficacy of existing vaccines highlights the need for developing effective, tailored vaccines, and further research is required to establish optimal vaccination protocols. Future conservation practices should integrate longitudinal serological surveillance with molecular detection techniques to distinguish active infections from past exposures, track potential viral genetic evolution, and establish evidence-based biosecurity protocols. This study provides a critical epidemiological foundation for FPV control in giant pandas and offers insights into host–pathogen-environment interactions in endangered wildlife.

## Supplementary Information

**Additional file 1.** **Sampling frequency distribution among the 136 giant pandas.** This file contains a frequency table showing the number of sampling times per individual, as well as the distribution of individuals with or without transfer history.

**Additional file 2.** **Detailed sampling history of the 18 individuals with ≥ 4 sampling years.** This file lists the 18 giant pandas that were sampled in four or more different years. For each individual, the sampling years, transfer history, and the corresponding antibody resultsare provided. The data support the longitudinal and cross‑sectional analyses presented in the manuscript.

## Data Availability

All data generated or analysed during this study are included in this published article.
